# Emergency Department Blood Pressure Treatment and Outcomes in Adults Presenting with Severe Hypertension

**DOI:** 10.5811/westjem.18126

**Published:** 2024-07-17

**Authors:** Farhan Chaudhry, Eliana Small, Steven J. Korzeniewski, Dana Benyas, Lydia Ross, Alex B. Hill, Amit Vahia, Candace McNaughton, Phillip Levy, Joseph Miller

**Affiliations:** * Department of Surgery, Detroit Medical Center/Wayne State University School of Medicine, Detroit, Michigan; † Department of Family Medicine and Population Health Sciences, Wayne State University School of Medicine, Detroit, Michigan; ‡ Department of Emergency Medicine, Henry Ford Health and Michigan State University Health Sciences, Detroit, Michigan; § Department of Emergency Medicine, Baylor College of Medicine, Houston, Texas; ∥ Ascension St. John Hospital, Detroit, Michigan; ¶ Department of Emergency Medicine, University of Toronto, Toronto, Canada; # Department of Emergency Medicine, Wayne State university, Detroit, Michigan

## Abstract

**Background:**

Patients who present to the emergency department (ED) with severe hypertension defined as a systolic blood pressure (SBP) ≥180 millimeters of mercury (mm Hg) or diastolic (DBP) ≥120 (mm Hg) without evidence of acute end-organ damage are often deemed high risk and treated acutely in the ED. However, there is a dearth of evidence from large studies with long-term follow-up for the assessment of major adverse cardiovascular events (MACE). We conducted the largest study to date of patients presenting with severe hypertension to identify predictors of MACE and examine whether blood pressure at discharge is associated with heightened risk.

**Methods:**

We enrolled ED patients with a SBP of 180–220 mm Hg but without signs of end-organ damage and followed them for one year. The primary outcome was MACE within one year of discharge. Secondarily, we performed a propensity-matched analysis to test whether SBP ≤160 mm Hg at discharge was associated with reduced MACE at 30 days.

**Results:**

A total of 12,044 patients were enrolled. The prevalence of MACE within one year was 1,865 (15.5%). Older age, male gender, history of cardiovascular disease, cerebrovascular disease, diabetes, smoking, presentation with chest pain, altered mental status, dyspnea, treatment with intravenous and oral hydralazine, and oral metoprolol were independent predictors for one-year MACE. Additionally, discharge with an SBP ≤160 mm Hg was not associated with 30-day MACE-free survival after propensity matching (hazard ratio 0.99, 95% confidence interval 0.78–1.25, *P* = 0.92).

**Conclusion:**

One-year MACE was relatively common in our cohort of ED patients with severe hypertension without acute end-organ damage. However, discharge blood pressure was not associated with 30-day or one-year MACE, suggesting that BP reduction in and of itself is not beneficial in such patients.

Population Health Research Capsule What do we already know about this issue?

*Patients with worsening levels of uncontrolled blood pressure have increasing risk of long-term cardiovascular events.*

What was the research question?

*How common are major adverse cardiovascular events (MACE) within one year for patients discharged from the ED with severe hypertension?*

What was the major finding of the study?

*While 15.5% had a cardiovascular event within a year, we found no association with initial ED encounter for so-called “hypertensive urgency.”*

How does this improve population health?

*Severe hypertension in the absence of hypertensive emergency identifies patients at significant risk of MACE, but acute BP reduction in the ED may not be immediately beneficial.*

## INTRODUCTION

### Background

Hypertensive emergencies are a significant concern when patients present to the emergency department (ED) with severely elevated blood pressure (BP), defined as ≥180 millimeters of mercury (mm Hg) systolic or ≥120 mm Hg diastolic pressure.^1^ Nevertheless, very few of these patients have evidence of acute end-organ damage. (EOD) and often have only severely uncontrolled chronic hypertension (HTN).^2^ ^,^ ^3^ Severely elevated BP, but without EOD, is often called “hypertensive urgency.” There is significant long-term evidence regarding characteristics associated with increased major adverse cardiovascular events (MACE) for patients with chronically elevated BP, but there is a lack of long-term evidence regarding characteristics associated with increased MACE in those presenting with hypertensive urgency in the ED.^2^ Even without this long-term evidence, there is temptation and expectation to acutely lower BP. Recent retrospective ED studies suggest that there may be no immediate benefit from acutely reducing BP in the setting of severe hypertension without EOD.^4^ ^,^ ^5^ This could be because there is evidence to suggest a greater risk for severe adverse effects with antihypertensives in the setting of severely elevated BP without EOD.^5^ ^,^ ^6^ However, larger outcome data for reaching a lower target BP for this population prior to discharge is lacking.

### Rationale

Given that there are significantly more patients presenting to the ED with severe HTN without EOD, identifying characteristics associated with MACE in this cohort has become increasingly more important.^1^ ^,^ ^3^ ^,^ ^7^ Likewise, there is uncertainty regarding the value of reaching a significantly lower target pressure prior to discharge from the ED for this cohort. Assessing the impact of reaching a target BP on MACE would also provide clinical utility for emergency physicians.

### Objective

To address these uncertainties, we conducted the largest observational cohort study to date to identify risk factors for MACE at one year among patients discharged home from the ED who presented with severely elevated BP without EOD. We also tested whether targeting a lower systolic blood pressure (SBP) prior to discharge was associated with reduced MACE at 30 days in this cohort. Our hypothesis for our primary analysis was that patients with SBP between 180–220 mm Hg without documented EOD and treated with anti-hypertensives would have less MACE at one year. We used an SBP greater than 180 mm Hg as this is the cutoff for hypertensive crisis according to the American Heart Association (AHA).^8^ Our secondary hypothesis was that propensity-matched patients with an initial SBP between 180–220 mm Hg without documented evidence of EOD, but who were discharged with a lower target SBP (≤160 mm Hg), would have less MACE at 30 days. We used a SBP ≤160 mm Hg, as >160 mm Hg is the cutoff for Grade 2 hypertension and has been shown to be associated with a high risk of EOD.^8^

## METHODS

### Study Setting and Population

Subjects included patients between 18–90 years of age who presented to one of eight EDs in an integrated health system (Henry Ford Health System, Detroit, MI) for treatment of any medical condition between January 2014–July 2015. These EDs consisted of one tertiary-care academic teaching hospital, three community teaching hospitals, and four freestanding EDs. The ED locations served urban communities with low socioeconomic status as well as suburban, more affluent communities. We included ED encounters that resulted in discharge from the ED and did not result in admission or being placed in observation. Adult patient encounters were abstracted from the electronic health record (EHR) shared by all EDs included in the study (Epic Systems Corporation, Verona, WI). We incorporated all ED encounters for adults for initial abstraction. Of these encounters, we excluded patient encounters with SBP >220 mm Hg or below <180 mm Hg and encounters with missing or incorrectly coded patient variables. For this study we defined acute EOD as an entered clinical diagnosis at presentation of acute coronary syndrome, stroke, heart failure exacerbation, or acute kidney injury in the EHR.

### Study Design

The study was approved by the international review board (IRB) at Henry Ford Hospital System. Data collection included the patient’s first ED BP and discharge BP, demographic information, comorbidities, insurance status, tobacco use, and clinical presentation. We used chief complaint as the primary symptom for their visit. We also recorded whether they were referred to the ED primarily for hypertension (HTN). Comorbidities included history of cardiovascular disease, stroke, diabetes, and chronic kidney disease. We classified insurance status into insured (Medicare, Medicaid, commercial) or not insured. We also collected the median income for the ZIP code where each patient resided. The median incomes for each ZIP code were grouped into quartiles for subsequent analysis. The patient’s antihypertensive therapies during the visit were recorded.

We used a method criterion described by Worster et al.^9^ 1) Two data abstractors were trained with the study criteria described above;. 2) The inclusion and exclusion criteria used were as stated above. 3) Variables were defined prior to analysis. 4) We used a standard abstraction form. 5) Abstractor performance was based on the number of records screened and verified based on accuracy of incorporating the criteria in record selection. 6) Abstractors during abstraction of records were blinded to the hypothesis. 7) Interobserver reliability was discussed, 8) but it was was not measured due to low number of observers to perform a statistical test. 9) The health record database was identified. 10) Method of sampling was described previously. 11) Any missing variable data of preidentified variables of interest resulted in removal of the entire patient record from the study. 12) The IRB did review the study.

### Primary and Secondary Outcomes

Each patient was tracked in the health system’s EHR up to one year following the date of the patient’s first ED encounter. We considered patients to have the primary outcome, MACE, if they had a documented diagnosis of acute heart failure, acute stroke (hemorrhagic or ischemic), acute coronary syndrome, or death within one year following their ED visit. We selected a one-year follow-up period to increase the number of events and thereby improve statistical power given the sample size.

If a patient had more than one cardiovascular event in the 12-month period, we only counted their first event for the purpose of the primary study outcome. Documented MACE from Epic’s Care Everywhere network was also included, so that we would not miss any MACE if the patient presented to an outside healthcare system within the state of Michigan using the same EHR. This incorporates deaths identified by the state of Michigan; however, if an event occurred outside the state, then this could not be incorporated. The Care Everywhere network incorporates the EHRs of multiple care systems in the state of Michigan, and records created within these care systems are automatically uploaded and validated in Care Everywhere. However, outside records may have been missed and, thus, may be a limitation to the study.

The secondary outcome was time-to-MACE within 30 days from discharge: a) because events that occur near discharge are more plausibly causally related than are events that occur long after discharge; and b) to test for differences in instantaneous risk velocity (ie, hazard ratio [HR]) conditional on survival (ie, to identify factors that are associated with shorter intervals-to-MACE at 30 days).

### Primary Analysis and Secondary Statistical Analyses

We used descriptive statistics to assess baseline demographic and clinical characteristics of the cohort. Continuous variables were expressed as median interquartile range (IQR), while categorical data were expressed as frequencies and proportions (ie, %). The Mann-Whitney U-test and the chi-square test were used to examine differences in distributions and proportions, respectively. In the primary regression analysis, we used logistic regression to estimate magnitudes of association between patient characteristics and MACE at one year. We chose logistic regression because the symmetric nature of the odds ratio (OR) enables inferences about associations between antecedents and outcomes or vice versa, unlike relative risk.^10^ Symmetry is ideal given the potential for confounding by indication in non-randomized clinical studies; that is, only ORs allow inferences about treatment given outcome and vice versa.^10^ ^,^ ^11^ Potential confounders were defined a priori as factors that are associated with both an exposure and outcome whose adjustment alters the estimated magnitude of association by >10%. We employed a two-stage model selection strategy. Firstly, we selected all factors that were associated with discharge SBP and MACE both in our study and based on prior knowledge (ie, full model). Next, to reduce the number of covariables and potential impact of multicollinearity, we used an agnostic strategy that optimized model accuracy measured by partial area under the curve at a 10% fixed-false-positive rate across 1,000 bootstrap replicates of training (70%) and testing datasets (30%). When considering ZIP code characteristics, we included a random intercept to account for similarities among patients nested in the same geographic area.

In the secondary regression analysis, we used the Cox proportional hazards model to estimate the magnitude of association between patient factors at ED discharge and time-to-MACE at 30 days. Given the potential for confounding by indication, and because we wanted to examine the clinical relevance of discharge SBP, we performed a propensity-matched analysis to balance the distribution of potential confounders between study groups that had SBP ≤160 mm Hg or >160 mm Hg.^12^ We used the MatchIt R-package to find pairs of observations with similar propensity scores but that differ in treatment status.^13^ That is, the primary logistic regression model was used to derive propensity-matched groups; factors that nevertheless differed between propensity-matched groups were included in the final multivariable Cox model as potential confounders. In both the primary and secondary analyses, magnitudes of association (ie, OR and HR) with 95% confidence intervals (CI) that do not include the null estimate (ie, ‘1.0’) are statistically significant. All analysis was performed using R version 4.0.3 (R Foundation for Statistical Computing, Vienna, Austria).

## RESULTS

### Demographics and Clinical Characteristics

Of 222,028 ED encounters reviewed during the study period, 13,042 (5.8%) patients met inclusion criteria. There were 12,044 (92.4%) who had complete records and were used for subsequent analysis. The overall number of patients that had MACE within one year was 1,865 (15.5%), inclusive of 176 deaths (9.5%).

Table 1 demonstrates the overall demographic and clinical characteristics of the cohort. Univariate analysis found that patients with MACE within one year following ED discharge were significantly more likely to be older (ages 65–90, OR 6.66 [95% CI 5.44–8.24] *P* < 0.001), and male (779/1,865 [41.8%] vs 3,719/10,179 [36.5%]; OR 1.25 [1.13–1.38], *P* < 0.001), but less likely to be Black (646/1.865[34.6%] vs 3,814/10,179 [37.5%]; OR 0.89 [0.80–0.98], *P* < 0.05). Also significantly associated with MACE within one year were history of cardiovascular disease (495/1,865 [26.5%] vs 251/10,179 [2.47%], OR 14.3 [13.2–16.8], *P* < 0.001); cerebrovascular disease (202/1,865 [10.8%] vs 136/10,179 [1.33%], OR 8.97 [7.18–11.2], *P* < 0.001); diabetes mellitus (607/1,865 [32.5%] vs 1,092/10,179 [10.7%], OR 4.02 [3.58–4.51], *P* < 0.001); and chronic kidney disease (352/1,865 [18.9%] vs 337/10,179 [3.31%], OR 6.80 [5.80–7.97], *P* < 0.001). Patients who used tobacco, had insurance, and those who lived in a ZIP code with a median annual income of $54,973–$70,439 US (75% quartile) were more likely to have MACE within one year in comparison to those with less than $44,583 (25% quartile) (OR 2.59 [2.24–2.87], *P* < 0.001, 3.81 [2.78–5.39], *P* < 0.001, and 1.17 [1.02–1.34], *P* < 0.05, respectively).

**Table 1.** Patient characteristics and univariate logistic regression comparisons for outcomes.

Variable	Total	No 1-year MACE	1-year MACE	OR (95% CI)	*P*-value
	12,044	10,179/12,044 (84.5%)	1,865/12,044 (15.5%)		
Demographic					
Age range in years, n(%)					
18–45	2,243/12,044 (18.6%)	2,139/10,179 (21.0%)	104/1,865 (5.58%)		
46–65	4,995/12,044 (41.5%)	4,409/10,179 (43.3%)	586/1,865 (31.4%)	2.74 (2.22–3.41)	*P* < 0.001
65–90	4,806/12,044 (39.9%)	3,631/10,179 (35.7%)	1,175/1,865 (63.0%)	6.66 (5.44–8.24)	*P* < 0.001
Male, n(%)	4,498/12,044 (37.3%)	3,719/10,179 (36.5%)	779/1,865 (41.8%)	1.25 (1.13–1.38)	*P* < 0.001
Black, n(%)	4,460/12,044 (37.0%)	3,814/10,179 (37.5%)	646/1,865 (34.6%)	0.89 (0.80–0.98)	*P* < 0.05
Medical history					
Cardiovascular disease, n(%)	746/12,044 (6.19%)	251/10,179 (2.47%)	495/1,865 (26.5%)	14.3 (12.2-16.8)	*P* < 0.001
Cerebrovascular disease, n(%)	338/12,044 (2.81%)	136/10,179 (1.33%)	202/1,865 (10.8%)	8.97 (7.18–11.2)	*P* < 0.001
Diabetes mellitus, n(%)	1,699/12,044 (14.1%)	1,092/10,179 (10.7%)	607/1,865 (32.5%)	4.02 (3.58–4.51)	*P* < 0.001
Chronic kidney disease, n(%)	689/12,044 (5.72%)	337/10,179 (3.31%)	352/1,865 (18.9%)	6.80 (5.80–7.97)	*P* < 0.001
Social history					
Tobacco smoker, n(%)	3,176/12,044 (26.3%)	2,358/10,179 (23.1%)	818/1,865 (43.8%)	2.59 (2.24–2.87)	*P* < 0.001
Has insurance, n(%)	11,258/12,044 (93.5%)	9,431/10,179 (92.7%)	1,827/1,865 (98.0%)	3.81 (2.78–5.39)	*P* < 0.001
ZIP code median annual income range in $US					
Less than 44,583, n(%)	4,663/12,044 (38.7%)	3,979/10,179 (39.1%)	684/1,865 (36.7%)		
44,584–54,972, n(%)	1,857/12,044 (15.4%)	1,549/10,179 (15.2%)	308/1,865 (16.5%)	1.16 (1.00–1.34)	0.05
54,973–70,439, n(%)	2,634/12,044 (21.9%)	2,192/10,179 (21.5%)	442/1,865 (23.7%)	1.17 (1.02–1.34)	*P* < 0.05
Over 70,440, n (%)	2,889/12,044 (24.0%)	2,459/10,179 (24.2%)	431/1,865 (23.1%)	1.02 (0.89–1.16)	0.77
Presentation					
Headache, n (%)	398/12,044 (3.31%)	346/10,179 (3.41%)	52/1,865 (2.79%)	0.81 (0.60–1.08)	0.70
Chest pain (%)	990/12,044 (8.22%)	799/10,179 (7.86%)	191/1,865 (10.2%)	1.34 (1.13–1.58)	*P* < 0.001
Altered mental status (%)	92/12,044 (0.772%)	53/10,179 (0.530%)	39/1,865 (2.09%)	4.01 (2.63–6.05)	*P* < 0.001
Dyspnea (%)	465/12,044 (3.86%)	329/10,179 (3.23%)	136/1,865 (7.29%)	2.36 (1.91–2.89)	*P* < 0.001
Referral for hypertension (%)	781/12,044 (6.48%)	675/10,179 (6.63%)	106/1,865 (6.02%)	0.85 (0.68–1.04)	0.13
Systolic blood pressure range in mm Hg (%)					
180–200	9,572/12,044 (79.5%)	8,158/10,179 (80.1%)	1,414/1,865 (75.8%)		
201–220	2,472/12,044 (20.5%)	2,021/10,179 (19.9%)	451/1,865 (24.2%)	1.288 (1.15–1.45)	*P* < 0.001
Diastolic blood pressure range in mm Hg (%)					
Less than 80	1,530/12,044 (12.7%)	1,182/10,179 (11.6%)	348/1,865 (18.6%)		
80–100	6,600/12,044 (54.8%)	5,577/10,179 (54.8%)	1,023/1,865 (54.9%)	0.62 (0.54–0.72)	*P* < 0.001
Greater than 101	3,914/12,044 (32.5%)	3,420/10,179 (33.6%)	494/1,865 (26.5%)	0.49 (0.42–0.57)	*P* < 0.001
Treatment	3,528/12,044 (29.3%)	2,345/10,179 (23.0%)	1,183/1,865 (63.3%)	5.80 (5.22–6.44)	*P* < 0.001
Clonidine oral (%)	1,220/12,044 (10.1%)	897/10,179 (8.81%)	323/1,865 (17.3%)	2.17 (1.89–2.49)	*P* < 0.001
Enalaprilat IV (%)	114/12,044 (0.946%)	74/10,179 (0.727%)	40/1,865 (2.14%)	2.99 (2.01–4.39)	*P* < 0.001
Labetalol oral (%)	241/12,044 (2.00%)	159/10,179 (1.56%)	82/1,865 (4.40%)	2.90 (2.20–3.79)	*P* < 0.001
Labetalol IV (%)	827/12,044 (6.87%)	559/10,179 (5.49%)	268/1,865 (14.4%)	2.89 (2.47–3.37)	*P* < 0.001
Metoprolol oral (%)	1,603/12,044 (13.3%)	809/10,179 (7.95%)	794/1,865 (42.6%)	8.59 (7.64–9.65)	*P* < 0.001
Metoprolol IV (%)	316/12,044 (2.62%)	174/10,179 (1.71%)	142/1,865 (7.61%)	4.74 (3.77–5.95)	*P* < 0.001
Hydralazine oral (%)	655/12,044 (5.44%)	319/10,179 (3.13%)	336/1,865 (18.0%)	6.79 (5.78–7.99)	*P* < 0.001
Hydralazine IV (%)	1,086/12,044 (9.01%)	656/10,179 (6.4%)	430/1,865 (23.1%)	4.35 (3.81–4.97)	*P* < 0.001
Outcome					
Discharge systolic blood pressure ranges in mm Hg (%)					
90–160	4,356/12,044 (36.2%)	3,641/10,179 (35.8%)	715/1,865 (38.3%)		
160–220	7,688/12,044 (63.8%)	6,538/10,179 (64.2%)	1,150/1,865 (61.6%)	0.90 (0.81–0.99)	*P* < 0.05
Acute coronary syndrome (%)	1,190/12,044 (9.88%)		1,190/1,865 (63.8%)		
Heart failure admission (%)	705/12,044 (5.85%)		705/1,865 (37.8%)		
Stroke (%)	488/12,044 (4.05%)		488/1,865 (26.2%)		
Death (%)	176/12,044 (1.46%)		176/1,865 (9.47%)		

*MACE*, major adverse cardiac event; *OR*, odds ratio; *CI*, confidence interval; *mm HG*, millimeters of mercury; *IV*, intravenous.

### Symptoms and Presenting BP

Patients presenting with chest pain, altered mental status, dyspnea and a SBP of 201–220 instead of 180–200 mm Hg were significantly more likely to suffer MACE (OR 1.34 [1.13–1.58], *P* < 0.001, 4.01[2.63–6.05], *P* < 0.001, 2.36 [1.91–2.89], *P* < 0.001, and 1.29 [1.15–1.45], *P* < 0.001), respectively. A chief complaint of headache or referral for HTN was not significantly associated with MACE (OR 0.81 [0.60–1.08], *P* = 0.17 and OR 0.85 [0.68–1.04], *P* = 0.13). Patients with a presenting diastolic blood pressure ranging from 80–100 mm Hg or 101 mm Hg and greater were less likely to suffer MACE (OR 0.62 [0.54–0.72], *P* < 0.001; 0.49 [0.42–0.57], *P* < 0.001).

### Treatment and Outcomes

There were 3,528/12,044 (29.3%) patients who had any antihypertensive treatment during their ED visit, and this treatment was significantly associated with a higher rate of MACE within one year compared to those who did not receive antihypertensive treatment (OR 5.80, 95% CI 5.22–6.44). Univariate analysis for each type of antihypertensive medication demonstrated that each was associated with MACE at one year. Furthermore, patients who were discharged with a SBP above 160–220 mm Hg were less likely to suffer MACE at one year (OR 0.90, 95% CI 0.81–0.99, Table 1).

### Primary Analysis

To create a parsimonious multivariable logistic regression model, the bootstrap receiver operating curve model retained age, gender, history of cardiovascular disease, cerebrovascular disease, diabetes mellitus, chronic kidney, smoking status, presentation with chest pain, altered mental status, dyspnea, treatment with hydralazine (oral and IV), oral metoprolol, and discharge SBP as variables (Supplement S1). The before-variable selection AUC was 0.842, and the after-variable selection AUC 0.839. Given that the estimated variance from the random effect of patient ZIP codes was low (0.0759, SD 0.275), this random effect was not included in our final analysis. In the adjusted model, older age range, male gender, history of cardiovascular disease, cerebrovascular disease, diabetes mellitus, smoking, presentation with chest pain, altered mental status, treatment with IV and oral hydralazine, and oral metoprolol were significant independent predictors for MACE at one year (Table 2). The model had a pseudo-R^2^ of 0.260. Retrospective power analysis performed with GPower 3 (https://www.psychologie.hhu.de/arbeitsgruppen/allgemeine-psychologie-und-arbeitspsychologie/gpower.html) showed a statistical power of this study over 90%.^14^

**Table 2.** Multivariable logistic regression of patient characteristics with a major adverse cardiac event (MACE) at one-year vs those without a MACE.

	OR (95% CI)	*P*-value
Age range in years		
18–45		
46–65	1.90 (1.53–2.39)	*P* < 0.001
65–90	4.04 (3.27–5.04)	*P* < 0.001
Male	1.35 (1.19–1.52)	*P* < 0.001
History of cardiovascular disease	4.62 (3.78–5.66)	*P* < 0.001
History of cerebrovascular disease	4.62 (3.48–6.15)	*P* < 0.001
History of diabetes mellitus	1.64 (1.41–1.91)	*P* < 0.001
History of chronic kidney disease	1.19 (0.96–1.48)	0.11
Tobacco smoker	1.35 (1.19–1.54)	*P* < 0.001
Chest pain	1.38 (1.13–1.68)	*P* < 0.01
Altered mental status	3.27 (1.92–5.49)	*P* < 0.001
Dyspnea	1.47 (1.11–1.93)	*P* < 0.001
Treated with hydralazine IV	1.98 (1.67–2.35)	*P* < 0.001
Treated with hydralazine oral	2.15 (1.74–2.65)	*P* < 0.001
Treated with metoprolol oral	4.88 (4.26–5.59)	*P* < 0.001
Discharge systolic blood ranges in mm Hg		
90–160		
161–220	0.94 (0.83–1.06)	0.295

Multivariate logistic regression results after achieving parsimony with ROC-bootstrap method.

*CI*, confidence interval; *IV*, intravenous; *mm Hg*, millimeters of mercury.

### Secondary Analysis

We compared short-term outcome (30-day MACE-free survival) for patients who were discharged with a SBP ≤160 mm Hg vs those with SBP >160 mm Hg. There were significant group differences for age, gender, history of cerebrovascular disease, smoking, presentation of headache, chest pain, presenting SBP, and reception of antihypertensive treatments between those who were discharged with a SBP ≤160 mm Hg compared to those with a SBP >160 mm Hg (Table 3).

**Table 3.** Pre- vs post-propensity score matching patient characteristics between patients discharged with a systolic blood pressure ≤160 vs >160.

Group characteristics for secondary analysis	Pre-match			Post-match		
Variable	Discharge SBP >160 mm Hg	Discharge SBP ≤160 mm Hg	*P-*Value	Discharge SBP >160 mm Hg	Discharge SBP ≤160 mm Hg	*P*-value
n	7,688	4,356		4,356	4,356	
Median age in years (IQR)	62 (50–74)	60 (49–72)	*P* < 0.001	60 (48–72)	60 (49–72)	0.36
Male (%)	2,954/7,688 (38.4%)	1,544/4,356 (35.4%)	*P* < 0.001	1,522/4.356 (34.7%)	1,544/4,356 (35.4%)	0.62
History of cardiovascular disease (%)	477/7,688 (6.2%)	269/4,356 (6.18%)	0.949	277/4,356 (6.29%)	269/4,356 (6.18%)	0.72
History of cerebrovascular disease (%)	198/7,688 (2.58%)	140/4,356 (3.21%)	*P* < 0.05	145/4,356 (3.26%)	140/4,356 (3.21%)	0.76
History of diabetes mellitus (%)	1,086/7,688 (14.1%)	614/4,356 (14.1%)	0.979	597/4,356 (13.4%)	614/4,356 (14.1%)	0.60
History of chronic kidney disease (%)	458/7,688 (5.97%)	231/4,356 (5.30%)	0.132	209/4,356 (5.07%)	231/4,356 (5.30%)	0.28
Tobacco smoker (%)	1,972/7,688 (25.7%)	1,204/4,356 (27.6%)	*P* < 0.05	1,289/4,356 (28.0%)	1,204/4,356 (27.6%)	0.72
Headache (%)	223/7,688 (2.90%)	176/4,356 (4.03%)	*P* < 0.01	212/4,356 (4.82%)	176/4,356 (4.04%)	0.06
Chest pain (%)	422/7,688 (5.48%)	569/4,356 (13.0%)	*P* < 0.001	421/4,356 (9.69%)	569/4,356 (13.1%)	*P* < 0.001
Altered mental status (%)	56/7,688 (0.73%)	37/4,356 (0.85%)	0.476	45/4,356 (0.90%)	37/4,356 (0.85%)	0.38
Dyspnea (%)	229/7,688 (2.98%)	236/4,356 (5.41%)	*P* < 0.001	228/4,356 (5.23%)	236/4,356 (5.42%)	0.70
Presenting median SBP in mm Hg (IQR)	190 (184–199)	190 (184–198)	*P* < 0.001	189 (184–198)	190 (184–198)	0.45
Received antihypertensive treatment (%)	2,158/7,688 (28.1%)	1,370/4,356 (31.5%)	*P* < 0.001	1,390/4,356 (30.6%)	1,370/4,356 (31.5%)	0.65

Pre- vs post-propensity demographics, presentation characteristics and outcomes. *T*-test comparisons are shown.

*SBP*, systolic blood pressure; *mm Hg*, millimeters of mercury; *IQR*, interquartile range,

After propensity-score matching, 4,356 controls with SBP >160 mm Hg and 4,356 patients with SBP ≤160 mm Hg were matched. Chest pain was the only variable that remained significantly different between patients with SBP >160 mm Hg vs those with SBP <160 mm Hg. This was included as a variable for adjustment for the following Cox regression analysis for 30-day MACE (Table 4). In the propensity-score matched analysis a chief complaint of chest pain was independently associated with an increased risk of 30-day MACE (HR 1.76, 95% CI 1.30–2.37, *P* < 0.001, Table 4).

**Table 4.** Cox regression analysis for 30-day MACE^*^-free survival after propensity matching.

Variable	30 day-MACE HR (95% CI)	*P*-value
Chest pain	1.76 (1.30–2.37)	*P* < 0.001
Discharge SBP ≤160	0.99 (0.78–1.25)	0.92

Cox regression analysis for 30-day MACE-free survival; discharge SBP≤160 variable is adjusted for chest pain as presenting clinical symptom after propensity score matching.

* *MACE*, major adverse cardiac event; *HR*, hazard ratio; *CI*, confidence interval; *SBP*, systolic blood pressure.

**Figure 1.**
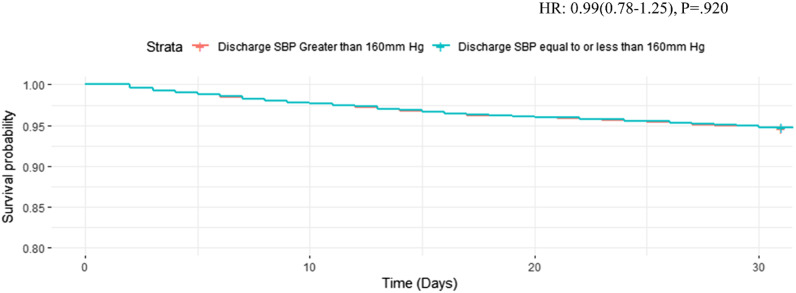
30-day MACE*-free survival curve for discharging patient with an SBP ≤160 after propensity score matching and adjusting for chest pain as presenting clinical symptom. *MACE, major adverse cardiac event; *HR*, hazard ratio; *SBP,* systolic blood pressure; *mm Hg*, millimeters of mercury.

However, discharge with an SBP ≤160 mm Hg was not associated with 30-day MACE-free survival after adjusting for covariates (HR 0.99, 95% CI 0.78–1.25, *P* = 0.92). Survival curve shows no significant survival benefit for patients who were discharged with a SBP ≤160 mm Hg vs those discharged with SBP >160 mm Hg (Figure 1).

## DISCUSSION

This study represents a large cohort of ED patients with severely elevated BP without EOD. There were high MACE rates at one year (15.5%) in our cohort. Independent risk factors for MACE at one year after an ED visit where severely elevated BP was noted included advanced age, male gender, history of cardiovascular disease, cerebrovascular disease, diabetes mellitus, and smoking. Presenting signs of chest pain and altered mental status, but not headache, were also found to be independent risk factors for MACE. Treatment with hydralazine or metoprolol was associated with higher rates of MACE at one year as well. Similar to findings by Patel et al, we found that referral to the ED for BP management by a clinic was not associated with an increased risk of MACE at one year.^4^

We used propensity-matched analysis to measure the association between reducing SBP in the ED and more immediate, 30-day MACE-free survival. In this analysis, there was no significant association between a reduction in SBP ≤160 mm Hg upon ED discharge and 30-day MACE. This was found even after propensity matching for reception of antihypertensive agents. This data adds to prior observational studies showing no associated benefit with a target BP prior to discharge from the ED.^5^

There was an association with certain antihypertensive treatments in the ED and higher one-year MACE. While it is unlikely that this antihypertensive treatment led to higher rates of MACE up to one year following treatment, it is plausible that clinicians perceive a clinical need to treat BP or heart rate immediately in patients with higher risk for MACE. This finding coincides with our previous work illustrating that severely hypertensive patients who were at higher risk for MACE were more likely to be given medications that quickly lower BP in the ED, such as nitrates, clonidine, hydralazine, metoprolol, labetalol, enalapril, and nicardipine, in addition to continuing their chronic HTN medications.^5^ As recent data shows an association with inpatient antihypertensive treatment and higher rates of acute kidney injury at 30 days, future work may consider this outcome in assessing the practice of acute BP lowering in the ED.^15^

Of note, we found no association between Black race and one-year MACE in patients with severe HTN on adjusted analyses. Studies have previously demonstrated significant association between Black race and increased incidence of MACE, attributing causation to higher prevalence of cardiovascular comorbidities, genetics, lower socioeconomic status, and different treatment received during hospitalization.^16^ ^,^ ^17^ Severe HTN is more common in Blacks and is present at a younger age in comparison to other races; therefore, there may be a protective bias among this population. It is important to note, however, that chronic HTN is different from an acute presentation of severe HTN, in which it is well established that Blacks have significantly worse outcomes.^18^

In our cohort, chest pain, altered mental status, and dyspnea were three chief complaints independently associated with one-year MACE. Prior data has indicated that patients who present to the ED with chest pain, with or without elevated BP, have about a 5% chance of developing MACE at one year.^19^ Of the 990 patients in this study who presented with a chief complaint of chest pain and had severely elevated BP, 191 (19.3%) developed MACE at one year. Of the 92 patients who presented with altered mental status, 39 (42.4%) developed MACE. Additionally, of the 465 patients who presented with dyspnea, 136 (29.2%) developed MACE. The combination of severely elevated BP and these complaints may indicate poor control of comorbid conditions, which contributes to vascular endothelial dysfunction and acute end-organ injury. While headache in the setting of severely elevated BP is considered by some to be indicative of a hypertensive emergency, it is notable that we found no association between this and MACE in our study.

### Clinical Perspectives

While hypertensive emergencies require emergent treatment, best practices remain controversial when patients have severely elevated BP and lack acute EOD.^1^ Often termed “hypertensive urgency,” a misnomer that implies some type of urgent intervention is needed, current guidance for management of such patients is largely based on expert opinion and varies significantly.^20^ Observational data shows no associated benefit for outpatient clinic referral to the ED for asymptomatic severe HTN.^4^ ^,^ ^21^ Guidance from the American College of Emergency Physicians recommends against antihypertensive therapy in the ED for patients with asymptomatic severe BP elevation.^22^ While some have advocated that the potential for developing EOD is concerning enough with severely elevated BP to justify urgent management in the ED, particularly in populations with poor ED follow-up care, there is no definitive evidence that intervening in such a manner improves short- or long-term outcomes.^20^ In fact, our study provides new evidence suggesting that the increase in long-term risk of MACE in this cohort is mostly due to established cardiovascular risk factors. Reaching a lower target SBP does not appear to be beneficial. While observational studies support this conclusion, a randomized clinical study is warranted to help confirm this statement. Management of severely elevated BP should be reserved for the outpatient setting. We identified key predictors for mortality in this population, and efforts should concentrate on better outpatient management of patients with these characteristics.

## LIMITATIONS

An important limitation to this study is the potential for unmeasured confounders that may have affected outcomes. Neither did we account for prior medication adherence or newly diagnosed HTN, factors that may impact a clinician’s approach to treatment in the ED.^5^ ^,^ ^21^ Another limitation is the lack of longitudinal follow-up BP and antihypertensive medication use over one year following the ED visit. While other published reports indicate that ED patients with severely elevated BP often continue to have uncontrolled BP, we did not collect such longitudinal data.^4^ Furthermore, we failed to collect potentially relevant biomarkers such as troponin and B-type natriuretic peptide to more accurately define patients with EOD. Thus, it is possible that clinicians may have missed diagnosing patients who may have been suffering from EOD. Events that occurred outside this health system, which were not documented in the Care Everywhere network, were not captured, and patients may have suffered subclinical or unreported events. It is unknown how many hospitals are registered in the Care Everywhere network. Together, these considerations may have led to an underestimation of the total number of events.

## CONCLUSION

Our study shows that the risk of subsequent major adverse cardiovascular events in ED patients with severely elevated blood pressure without end-organ damage is high. Patients at higher risk of MACE often suffer from well-established cardiovascular risk factors. While these patients stand to benefit from carefully coordinated follow-up for cardiovascular risk reduction, reaching a target BP in the ED is not associated with improved outcomes. This data adds to mounting evidence against the utility of having a target BP prior to discharge from the ED and suggest that the term “hypertensive urgency” be avoided to describe such individuals, as there is no need for urgent intervention.

## Supplementary Information



