# Supplementary material for: Emergency Department Blood Pressure Treatment and Outcomes in Adults Presenting with Severe Hypertension
Source: West J Emerg Med. 2024 Jul 17;25(5):680–9. doi: 10.5811/westjem.18126 (PMC11418874; doi:10.5811/westjem.18126)

**Supplementary Figure**

**S1: ROCs Before and After Variable Selection**

AUC for the ROCs both before and after variable selection.


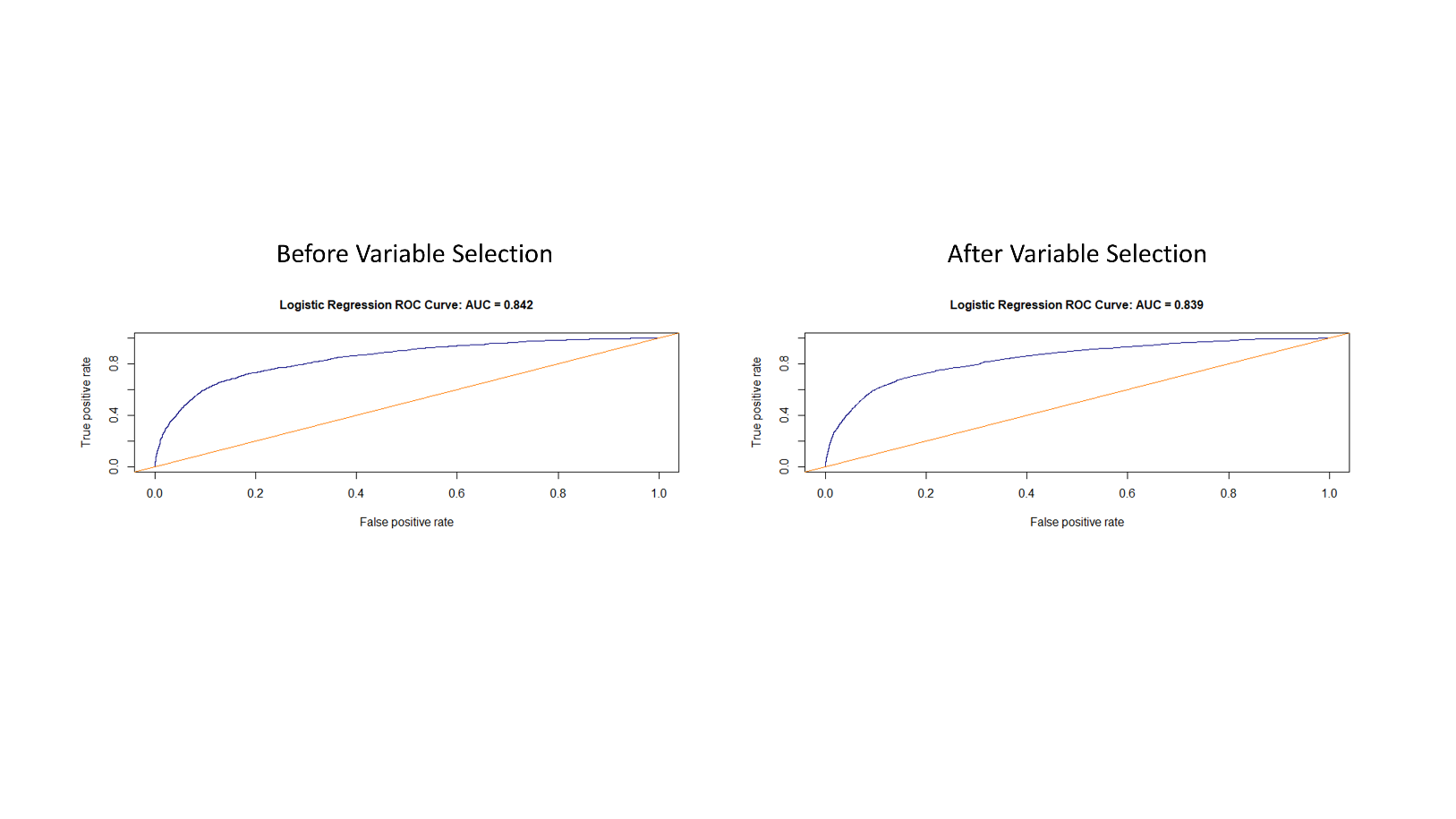

Supplement: Supplementary file 1 [file wjem-25-680-s001.docx]
